# Mozambique’s journey toward accreditation of the National Tuberculosis Reference Laboratory

**DOI:** 10.4102/ajlm.v6i2.491

**Published:** 2017-03-31

**Authors:** Sofia O. Viegas, Khalide Azam, Carla Madeira, Carmen Aguiar, Carolina Dolores, Ana P. Mandlaze, Patrina Chongo, Jessina Masamha, Daniela M. Cirillo, Ilesh V. Jani, Eduardo S. Gudo

**Affiliations:** 1 Instituto Nacional de Saúde, Ministério da Saúde, Maputo, Mozambique; 2 Centers for Disease Control and Prevention, Maputo, Mozambique; 3 IRCCS San Raffaele Scientific Institute, WHO Supranational TB Reference Laboratory, Tuberculosis & Mycobacteria Unit, Milan, Italy

## Abstract

**Background:**

Internationally-accredited laboratories are recognised for their superior test reliability, operational performance, quality management and competence. In a bid to meet international quality standards, the Mozambique National Institute of Health enrolled the National Tuberculosis Reference Laboratory (NTRL) in a continuous quality improvement process towards ISO 15189 accreditation. Here, we describe the road map taken by the NTRL to achieve international accreditation.

**Methods:**

The NTRL adopted the Strengthening Laboratory Management Toward Accreditation (SLMTA) programme as a strategy to implement a quality management system. After SLMTA, the Mozambique National Institute of Health committed to accelerate the NTRL’s process toward accreditation. An action plan was designed to streamline the process. Quality indicators were defined to benchmark progress. Staff were trained to improve performance. Mentorship from an experienced assessor was provided. Fulfilment of accreditation standards was assessed by the Portuguese Accreditation Board.

**Results:**

Of the eight laboratories participating in SLMTA, the NTRL was the best-performing laboratory, achieving a 53.6% improvement over the SLMTA baseline conducted in February 2011 to the Stepwise Laboratory Quality Improvement Process Towards Accreditation (SLIPTA) assessment in June 2013. During the accreditation assessment in September 2014, 25 minor nonconformities were identified and addressed. In March 2015, the NTRL received Portuguese Accreditation Board recognition of technical competency for fluorescence smear microscopy, and solid and liquid culture. The NTRL is the first laboratory in Mozambique to achieve ISO 15189 accreditation.

**Conclusions:**

From our experience, accreditation was made possible by institutional commitment, strong laboratory leadership, staff motivation, adequate infrastructure and a comprehensive action plan.

## Introduction

Implementation of quality management systems (QMS) ensures that laboratory services meet international standards and that results are accurate, reliable and timely, representing a vital role in diagnosis, monitoring of disease treatment, training, surveillance and disease prevention.^1^ However, in resource-limited settings, laboratories are poorly funded and suffer from chronic underinvestment, resulting in inadequate infrastructure, lack of equipment maintenance and calibration services, irregular training for laboratory workers and lack of QMS.^2,3^ To improve the quality of laboratory services in Africa, the World Health Organization’s Regional Office for Africa and its international partners established a framework that allows for incremental implementation of QMS. This process, called the Stepwise Laboratory Quality Improvement Process Towards Accreditation (SLIPTA) recognises laboratories’ efforts at each level toward full implementation of the ISO 15189 Standard requirements using a zero- to five-star grading system.^4^ In 2011, the Mozambique Ministry of Health adopted this approach and implemented the first round of Strengthening Laboratory Management Toward Accreditation (SLMTA), an innovative training and mentorship programme for continuous quality improvement, with the aim of improving patient care.^5,6^ The National Tuberculosis Reference Laboratory (NTRL) was one of the eight participating laboratories enrolled in SLMTA.

Established in 1987, the NTRL falls under the Mozambique National Institute of Health (MNIH), within the Ministry of Health. The MNIH’s mission is to contribute to the improvement of the well-being of the people of Mozambique by generating and promoting scientific and technological solutions to the country’s principal health issues.^7^ Beside the NTRL, the MNIH hosts nine other reference laboratories, including two regional tuberculosis reference laboratories that are overseen by the NTRL, and reference laboratories for cellular immunology, molecular virology, virus isolation, parasitology, entomology, microbiology and serology.

The NTRL provides laboratory reference services for the entire national health system in Mozambique. These include diagnosis of tuberculosis, multi-drug resistant tuberculosis and extensively drug resistant tuberculosis and treatment monitoring and support of the laboratory network through training activities, technical assistance through on-site supervision, and provision of proficiency panels for external quality assessment. The NTRL also conducts surveillance for tuberculosis drug resistance and research activities. These services must be of high quality and meet international standards.

In this article, we describe the NTRL’s road map, challenges and successes during the journey taken from February 2011 to March 2015 to attain international accreditation.

## Methods

### Setting

**FIGURE 1:**
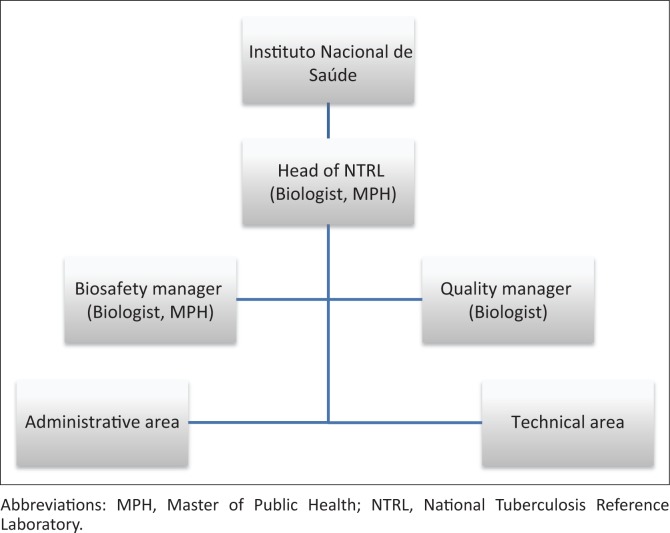
National Tuberculosis Reference Laboratory organizational structure, Mozambique.

The NTRL is in the city of Maputo.^7^ At the time of accreditation, the NTRL had 18 staff members, including 10 biologists, four of whom had Masters degrees, four laboratory technicians, two administrative staff and two cleaning staff (Figure 1). The laboratory has an average workload of 50 samples per day, and performs tuberculosis culture in solid and liquid media, fluorescence smear microscopy, first- and second-line drug susceptibility testing (DST), acid-fast bacilli identification, identification of *Mycobacterium tuberculosis* complex by immunochromatography, and molecular (Cepheid GeneXpert^®^ MTB/RIF) and line-probe assays (Hain Genotype^®^ MTBDR*plus*).

### Infrastructure improvement

In 2009, the NTRL underwent renovation to improve biosafety and workflow. The renovation process was completed in 2010 and acted as a starting point in the process toward international accreditation. The renovation period was stressful for the laboratory staff as they twice had to move from one facility to another. To make matters worse, the NTRL was closed for four months to pave the way for renovations, which left the country without the reference services provided by the NTRL.

### Pre-SLMTA period

Prior to SLMTA training, none of the staff from NTRL had previous experience in QMS. No QMS had been implemented in the laboratory, documentation and records were poorly controlled, equipment maintenance and calibration were poorly monitored, corrective actions were not undertaken and staff were not conscious about the importance of implementing QMS. External proficiency panels were only available for smear microscopy and DST. Only four quality indicators were monitored on a yearly basis: (1) quality of sputum samples; (2) smear microscopy and culture positivity rate; (3) contamination rate; and (4) workload.

### MNIH strategy for achieving accreditation

**FIGURE 2:**
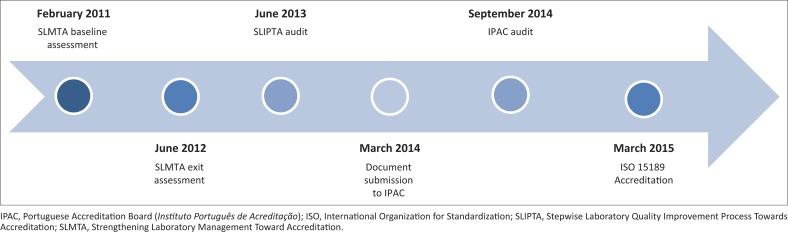
Timeline of events in the pathway to ISO 15189 accreditation, Mozambique, 2011–2015.

The MNIH used a four-pronged approach to achieve accreditation of the NTRL. These included the creation of the National Program for Laboratory Accreditation, the adoption of the SLMTA training pack, the SLIPTA assessment, and the mentorship from expert assessors. Some of the approaches were implemented concurrently (SLMTA, mentorship) and the SLIPTA assessment was used at the end, to measure progress and determine readiness for accreditation. Figure 2 outlines the timeline of key events in the pathway to accreditation.

### National Program for Laboratory Accreditation

To support QMS implementation, the Ministry of Health adopted SLMTA as its training strategy in 2011. To ensure institutionalisation and ownership of SLMTA in the health system, the Ministry of Health decided to translate the acronym of SLMTA into Portuguese and adopted the acronym FOGELA, a direct translation of SLMTA to Portuguese: ***FO**rtalecimento da **GE**stão de **L**aboratórios para **A**creditação*. The Ministry of Health also decided to establish the National Program for Laboratory Accreditation, which represented the backbone for implementation of SLMTA. The main mandate of the National Program for Laboratory Accreditation was to provide institutional leadership for SLMTA implementation at the Ministry of Health, as well as the framework for laboratory accreditation in Mozambique.

### NTRL participation in SLMTA training

Together with seven other laboratories from Mozambique, the NTRL participated in the first round of SLMTA implementation in Mozambique between February 2011 and June 2012. In total, five staff from NTRL participated in the SLMTA training as trainees. None of the NTRL staff members became SLMTA master trainers. A baseline audit by SLMTA trainers was conducted in February 2011, using Version 1 of the SLIPTA checklist. That checklist comprised 12 sections that are aligned with the 12 Quality System Essentials. A total score of 250 points was possible.^8^

Based on the recommendations and list of nonconformities from the baseline audit, an action plan was immediately drafted and approved. Furthermore, the NTRL completed the three SLMTA workshops, intercalated by a total of six site visits and six quality improvement projects that were a mandatory part of SLMTA. Table 1 presents the actions taken and outcomes for each improvement project. Internal meetings were conducted after each workshop to engage all laboratory staff in implementing the improvement projects, and an exit audit was conducted in March 2012.

**TABLE 1:** National Tuberculosis Reference Laboratory improvement projects during first round of Strengthening Laboratory Management Toward Accreditation, 2011–2013, Mozambique.

Improvement project	Objective	Actions taken	Outcome
Improve the quality of samples received	Reduce the number of samples rejected to less than 5%	Develop and distribute instructions for sample collection, packing and transportation Train NTRL staff, sample carriers and drivers Indicator monitored monthly	Rejection rate decreased from 27% to 4%
Improve temperature records for equipment	Monitor the temperature of all applicable equipment in the laboratory	Develop temperature monitoring forms Procure thermometers Train NTRL staff Monitor temperature deviations and apply corrective actions, if deviation is detected Indicator monitored monthly	Reagents and samples stored at adequate temperature
Improve turn-around times	Reduce the TAT of smear microscopy to 48 hours, solid culture to 62 days, and DST to 28 days	Develop a worksheet for each laboratory test to monitor TAT Validate results on the same day they are ready Train NTRL staff Indicator monitored monthly	TAT for smear microscopy reduced from 72 to 48 hours TAT for solid culture reduced from 66 to 62 days TAT for DST reduced from 32 to 29 days
Customer satisfaction	Measure customer satisfaction across the laboratory network	Survey implementation Train NTRL staff to apply the survey in several Health Units and for outpatients. Create a complaints book Analyse customer satisfaction survey and define improvement actions Survey performed twice a year	85% of customers were very satisfied with NTRL performance in the following survey
Personnel files	All staff must have their personnel files completed	Develop a list containing all documentation and information needed for each staff member Inform NTRL staff to provide all necessary documentation Files are updated frequently and reviewed on a yearly basis	90% of the staff personal files were completed
Equipment life books	Create life books for all laboratory equipment	Prepare registration forms, SOPs and folders for each piece of equipment Create a maintenance and calibration plan for each piece of equipment	All equipment calibrated and under a maintenance plan

Abbreviations: DST, drug susceptibility testing; NTRL, National Tuberculosis Reference Laboratory; SOP, standard operating procedures; TAT, turn-around time.

### Mentorship

Mentorship was provided at all stages of the preparation for the NTRL accreditation and included technical mentorship to strengthen capacity for diagnostic procedures. During the SLMTA training, a mentorship programme was designed to support the implementation of the quality improvement projects. The programme included six- to eight-week periods when the mentor was in the laboratory, followed by an eight-week absence. Supervisory visits were conducted to provide technical assistance, to monitor the implementation process and progress and to review laboratory action plans.

After the SLIPTA assessment, a mentor was assigned to support and guide the laboratory, working closely with the quality manager and head of the NTRL, in preparation for accreditation. The mentor had more than 15 years of experience in implementing QMS in private and public clinical laboratories in Portugal and, in line with the ISO 9001 and ISO 15189 standards, had a post-graduate degree in quality management and was also a senior certified auditor.

### SLIPTA audit

The NTRL was one of four laboratories selected by the National Program for Laboratory Accreditation to pursue a SLIPTA audit. The SLIPTA audit took place in June 2013 and was a two-day process conducted by the African Society for Laboratory Medicine’s certified external auditors (two expert international auditors and four locally-trained auditors). The updated Version 2 of the SLIPTA checklist based on 258 points was used.^9^ On Day 1, the auditors reviewed the laboratory’s policies, procedures and records for its competency, completeness and compliance with the ISO 15189:2012 standard. In addition to interviewing staff members, they also observed laboratory processes in the technical areas. On Day 2, the assessor team and the laboratory staff discussed all audit findings, including recommendations from the assessors on how to close identified gaps.

### Post-SLIPTA assessment and preparation for the Portuguese Accreditation Board audit

Following the SLIPTA audit, an action plan was drawn up to address the audit findings, a management review with the MNIH directorate was scheduled and several training activities were conducted. The training included ISO 15189:2012 standard requirements, biosafety, several standard operating procedures and sample transportation. Additional quality indicators were developed and/or monitored regularly for pre-analytical, analytical and post analytical phases. Crucial laboratory indicators that were regularly monitored (monthly or quarterly) included: (1) sample rejection rate; (2) nonconformities in request forms; (3) culture contamination rate; (4) concordance of results between smear microscopy and culture versus type of patients (new cases or previously treated); (5) laboratory turn-around time; (6) client satisfaction; and (7) workload.

Both internal (performed by the MNIH Quality Unit) and external audits (performed by an independent, external certified auditor) flagged the most challenging issues to be resolved as: (1) unavailability of certified external services and suppliers; (2) adequately maintaining laboratory equipment, reagents and consumables; (3) ensuring quality of examination results; and (4) post-examination processes (Table 2).

**TABLE 2:** Main challenges experienced and actions taken to accomplish ISO 15189:2012 requirements for accreditation of the National Tuberculosis Reference Laboratory, Mozambique, 2011–2013.

Requirements for quality and competence: ISO 15189:2012 international standards†	Critical points	Actions taken
4.6 External services and suppliers	In case of an emergency situation, difficulty in referring samples to an accredited laboratory located in Mozambique, since NTRL was the first to be accredited. Difficulty in defining other criteria that could be accepted by the accreditation board.	Select a laboratory with good performance on EQA results, have a written agreement, to be renewed annually, according to their EQA results.
5.3 Laboratory equipment, reagents, and consumables	Difficulties in selecting and hiring a local company to provide equipment service, which follows all ISO requirements for calibration (companies should be ISO 17025 accredited to perform relevant calibration of equipment, e.g., specific temperature ranges for incubators).	Select services from abroad that accomplish all requirements.
5.6 Ensuring quality of examination results	Difficulties in maintaining an EQA for culture for four consecutive years (accreditation cycle), including customs clearance of imported EQA schemes.	EQA for culture was purchased. An agreement with an EQA provider in South Africa and with a transportation company was signed, including transport and customs clearance.
5.7 Post-examination processes	The report of test results did not include all ISO 15189 requirements, due to constraints in adapting the LIS format.	Testing reports were redesigned according to ISO 15189 requirements.

Abbreviations: EQA, external quality assurance; ISO, International Standards Organization; LIS, laboratory information system; NTRL, National Tuberculosis Reference Laboratory.

† Other ISO 15189 requirements are not listed in the table, since they were not linked to the major critical points.

### ISO 15189 accreditation

In March 2014, a year after the SLIPTA audit, the NTRL decided to submit its application for an ISO 15189 accreditation assessment for fluorescence smear microscopy and culture in solid and liquid media to the Portuguese Accreditation Board (*Instituto Português de Acreditação*; IPAC). To apply, the NTRL followed all procedures, rules, criteria and methodology as described in IPAC’s General Regulations.^10^ For development of their accreditation activities, IPAC has Technical Committees that comprise evaluators and external experts, as well as a Consultative Committee that supervises the impartiality of all processes.

An appointed evaluation team examined the application to ensure that all required documentation was included. The application was then approved and the audit dates were scheduled. The assessment, conducted by two IPAC auditors (one for QMS and one technical), took place on 22–23 September 2014. Following the assessment, a report, including all non-conformities that had to be corrected for demonstration of compliance with the accreditation standards, was discussed and delivered to the laboratory team. Within six months all non-conformities had been resolved and evidence thereof was sent to IPAC.

### Costs associated with accreditation

From the beginning of the process, the MNIH directorate was committed to the accreditation, and efforts were made to ensure that sufficient resources and timely support were available to the process. Discussions were also conducted with several partners to provide additional support. The main costs were related to laboratory renovation, followed by technical assistance/mentorship and equipment maintenance (Table 3).

**TABLE 3:** Approximate costs of National Tuberculosis Reference Laboratory accreditation, Mozambique, 2011–2013.

Item	Approximate cost for accreditation (USD)	Approximate cost to maintain accreditation (USD)
Renovation of the laboratory and purchase of new equipment	1 500 000	60 000
Laboratory information system and informatics equipment	47 000	12 850 (LIS license)
Laboratory equipment maintenance/calibration (per year)	60 000	60 000
National and international training activities	50 000	50 000
Mentorship (per year)	84 000	60 000
Participation in EQA	100	100
System for laboratory access restriction and surveillance cameras	5000	900 (System maintenance)
Internal audits (External auditor from abroad)	6000	6000
ISO 15189 process (including airfare, accommodations for the audit team)	7800	7800
Emergency exit stairs	40 000	-
Back up electrical generator / Fuel	25 054	1500 (Fuel)
Negative pressure system	39 000	8000 (Maintenance of NPS)

**Total**	**1 863 954**	**207 150**

Abbreviations: EQA, external quality assessment; ISO, International Organization for Standardization; LIS, laboratory information system; NPS, negative pressure system; USD, United States dollars.

## Results

### SLMTA training programme

**FIGURE 3:**
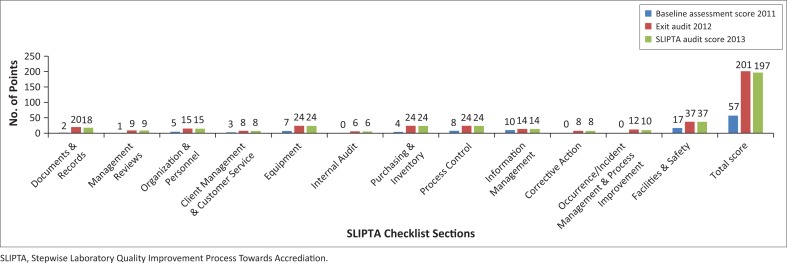
Percentage scores for National Tuberculosis Reference Laboratory performance in each section of the Stepwise Laboratory Quality Improvement Process Towards Accreditation checklist at baseline, exit and final Strengthening Laboratory Management Toward Accreditation audits.

Six quality improvement projects were conducted in the NTRL during the SLMTA training (Table 1). From baseline to exit audit, the NTRL improved its score from 57 (22.8%) to 201 (77.9%) points (Figure 3). Improvements were observed across all 12 checklist sections, and the highest scores were achieved in the *Facilities and Safety* and *Client Management and Customer Service* sections, which made the NTRL the best-performing laboratory among the eight laboratories in the first SLMTA round. The high performance of the NTRL in the exit audit led to its selection for a SLIPTA audit. Mentorship and supervision were professional and effective at equipping staff with the skills required for high quality performance.

### SLIPTA programme

During the SLIPTA audit, the laboratory scored 197 points (76.36%), corresponding to three stars, which was similar to the SLMTA exit audit scores (Figure 3).

### ISO 15189 accreditation

The IPAC assessment revealed 25 minor nonconformities (Table 2), most of which were related to document control. All nonconformities were addressed in a timely manner. On 27 March 2015, the NTRL was recognised by IPAC as having implemented a QMS and having the technical competency for fluorescence smear microscopy and solid and liquid culture. The NTRL thus became the first medical laboratory in Mozambique to achieve ISO 15189 accreditation.

## Discussion

Internationally-accredited laboratories are recognised for their superior test reliability, operational performance, quality management and competence.^11^ In pursuit of this recognition, the NTRL began its journey by first renovating the laboratory as a foundation for establishing QMS. The good infrastructure contributed to the high scores achieved for *Facilities and Safety*, an area that pulls down the scores of many laboratories in resource-limited settings.^2,12^ Moving to a newly-renovated facility was also motivating for all NTRL staff.

The starting point for implementing a QMS at the NTRL was the SLMTA training. The NTRL improvement during the SLMTA training was outstanding. Indeed, an evaluation of six of the eight laboratories participating in the first round of SLMTA showed that all had improved their scores.^13^ At baseline, all laboratories began with zero stars and at the SLIPTA audit, only one laboratory remained at zero stars (though its score still increased from 14% to 44%), three laboratories were at one star, one laboratory was at two stars and the NTRL reached three stars.^13^

NTRL participation in the SLMTA programme was important for the introduction of basic QMS concepts, and for demonstrating the importance of implementing QMS and its impact on patient care. Since several laboratories participated in the first SLMTA round, healthy competition between the laboratories was also a motivating factor, pulling each laboratory to work harder and better at implementing QMS towards achieving the highest number of stars.

Although NTRL management were aware of the existence of other resources for attaining accreditation (e.g., the Global Laboratory Initiative’s Stepwise Process towards TB Laboratory Accreditation),^14^ it was concluded that using different tools or checklists could disturb the team’s focus. Thus, the NTRL decided to concentrate on using the SLIPTA tool and the ISO 15189 standard as a foundation to meet international standards and achieve ISO 15189 accreditation.

The scores achieved at the SLIPTA assessment were slightly lower than the SLMTA exit assessment scores. One reason for that might be the use of a different checklist (version 2 versus version 1). Another reason could be that the evaluation performed by highly-experienced auditors during the SLIPTA audit was more rigorous.

The SLIPTA assessment increased staff motivation and confidence that ISO accreditation was an achievable goal. At that point, the entire team believed that the NTRL could become the first internationally-accredited medical laboratory in Mozambique. Staff members were motivated to work together as a team, to learn more, and to develop a comprehensive calendar and action plan for gradual implementation of quality improvement plans. Training further enhanced technical capacity and staff motivation to implement a QMS. However, maintaining constant staff motivation was a challenge and required continuous investment and innovation. For example, small rewards were given in appreciation of staff members who made outstanding contributions to the accreditation process. Building on this motivation, the MNIH leadership, with financial support from partners, identified an external mentor to support the laboratory in its pursuit, and from that moment on, a continuous mentorship programme replaced the intermittent mentorship of the SLMTA training. The mentor’s presence and expertise were essential in mentoring the process towards accreditation.

With regard to the ISO 15189 application to IPAC, IPAC was selected as the accrediting body, primarily because of language, since Mozambique is a Portuguese-speaking country. Not only did that facilitate communication between the audit team and the laboratory staff during and after the assessment, but it also negated the need for prior translation of QMS documents into English.

Since the NTRL was just beginning implementation of a QMS, the focus was on three basic assays to guarantee that the ISO requirements were well established for smear microscopy and culture in solid and liquid media. Additionally, during the SLMTA training, quality indicators for those assays were created and have been monitored regularly since then. Good results on external quality assurance (EQA) were also considered as a selection criterion. Recently, the NTRL submitted an application to expand its accreditation to include acid-fast bacilli identification, *Mycobacterium tuberculosis* complex identification by immunochromatography and GeneXpert^®^ MTB/RIF. The overall goal is to have all assays performed by the NTRL accredited by 2018.

During the accreditation process, internal and external audits were equally important for identifying areas that required attention and for informing the action plans. A clearly defined action plan mapped the pathway towards the goal, assigned responsibilities and built the platform for accountability toward achieving the common objectives, while keeping team members focused on activities and timelines. It was the experience of the NTRL that a very well-designed and comprehensive action plan was the key to successful implementation of a QMS and achieving accreditation.

Although African countries have relied much on external support to strengthen public health laboratories and other health systems,^15^ country leadership represents a critical factor for achieving accreditation.^3,16,17^ In the NTRL’s experience, commitment at all levels of the organisation played the most important role in improving and sustaining continuous laboratory quality. MNIH leadership not only provided financial support for the accreditation process but also participated actively in audit close-out meetings and the management review, as well as in engaging external partner support.

To commemorate this outstanding achievement, a public ceremony led by the Minister of Health was held on 13 April 2015. The Minister presented the accreditation certificate to the NTRL in the presence of civil society, implementing partners, several international agencies and stakeholders from the Ministry of Health and other public institutions.

Accreditation is a long journey that presents several challenges, especially in resource-poor countries. A major challenge experienced during the NTRL accreditation process included lack of local companies with proper expertise and certification to provide services for equipment maintenance and calibrations. Other challenges encountered, as well as solutions, are outlined in Table 2.

The main objective of having a QMS in place is to deliver quality laboratory services ensuring that patients receive timely and reliable results, thereby guaranteeing accurate diagnosis and treatment. With a QMS implemented, the laboratory continuously monitors daily operations and easily identifies areas that require more attention for continuous and systematic improvement. The regular monitoring of quality indicators allows for rapid identification of system weaknesses and rapid resolution of problems. The experience of ISO 15189 accreditation at the Kenya Medical Research Institute/Centre for Disease Control HIV-Research Laboratory in Kisumu, Kenya showed a reduction in reagent wastage, leading to increased cost savings to the laboratory, as a major benefit of implementing a QMS.^16^ Although such an analysis was not performed at the NTRL, stock management system was improved and became more effective, enabling accurate forecasting of needs and avoidance of stock outs and reagents expiring, thus minimising waste. The accreditation of the NTRL also increased research partner’s confidence in the laboratory’s results and has increased the number of research collaborations and projects undertaken. Implementation of a QMS enhanced staff competency, provided by enhanced performance on external quality proficiency and internal competency assessments (data not shown). On a daily basis, the accreditation demonstrates that the laboratory is more competent in its mission to improve the health system in Mozambique.

In the journey toward accreditation, some aspects such as understanding and routine implementation of document control procedures could have been approached differently. For instance, increased training of NTRL staff on key quality concepts such as ‘the meaning of quality’, ‘the cost of not implementing quality’, ‘the importance of procedures to detect errors’ and ‘client-focused service provision’; proper understanding of quality concepts and the benefits of implementing quality which influences maintenance of daily documentation procedures in the laboratory; and multiple and routinely-scheduled internal audits looking at a few requirements at a time might have improved timely identification and resolution of weaknesses in laboratory processes. We believe these approaches may have helped establish a continuous quality improvement culture in the laboratory and would guarantee that accreditation is not only achieved but maintained.

## Conclusion

Implementing a QMS is a continuous process that needs to be improved constantly in order to maintain accreditation. The benefits of implementing a QMS outweigh the challenges encountered and impact client and staff satisfaction, better service delivery and overall healthcare improvement. Many factors contributed to achieving international accreditation in Mozambique, including availability of financial resources, well-trained and motivated staff to implement the QMS and robust action plans. Most importantly, the commitment at all levels, especially from high-level leadership and stakeholders turned the dream of accreditation into a reality.

BOX 1 Lessons Learned.

- Commitment and support from institutional leadership and stakeholders is critical for achieving accreditation.
- Well-trained and motivated staff are required to implement any quality management system.
- Adequate funding and infrastructure, as well as a robust action plan, are necessary for successful pursuit of accreditation.
